# Diagnostic accuracy of procalcitonin in adult non-neutropenic cancer patients with suspected infection: a systematic review and meta-analysis

**DOI:** 10.1186/s12879-024-09174-7

**Published:** 2024-03-04

**Authors:** Yi-Chih Lee, Hsin-Tzu Yeh, Sz-Wei Lu, Yi-Chun Tsai, Yu-Chen Tsai, Chieh-Ching Yen

**Affiliations:** 1 Department of Emergency Medicine, Linkou Branch, Chang Gung Memorial Hospital, No. 5 Fushing St., Gueishan Shiang, Taoyuan, Taiwan; 2 Department of Emergency Medicine, Tri-Service General Hospital SongShan Branch, National Defense Medical Center, Taipei, Taiwan; 3 Department of Medicine, Critical Care, Queen Mary University of London, London, UK; 4 Department of Diagnostic Radiology, Chang Gung Memorial Hospital, Keelung, Taiwan; 5 Department of Emergency Medicine, New Taipei Municipal Tucheng Hospital, New Taipei City, Taiwan; 6 Institute of Emergency and Critical Care Medicine, National Yang Ming Chiao Tung University, Taipei, Taiwan

**Keywords:** Infection, Biomarker, Procalcitonin, meta-analysis

## Abstract

**Background:**

Procalcitonin (PCT) has garnered attention as a potential diagnostic biomarker for infection in cancer patients. We performed a systematic review and meta-analysis to evaluate the diagnostic accuracy of procalcitonin (PCT) and to compare it with C‐reactive protein (CRP) in adult non-neutropenic cancer patients with suspected infection.

**Methods:**

A systematic literature search was performed in MEDLINE, EMBASE, and Cochrane Central Register of Controlled Trials to identify all relevant diagnostic accuracy studies. Original articles reporting the diagnostic accuracy of PCT for infection detection in adult patients with solid or hematological malignancies were included. The pooled sensitivity, specificity, positive likelihood ratio, negative likelihood ratio, diagnostic odds ratio, area under the hierarchical summary receiver operator characteristic (HSROC) curve, and corresponding 95% confidence interval (CI) were calculated.

**Results:**

Seven studies were included in the meta-analysis. The pooled sensitivity and specificity of PCT were 60% (95% CI [45–74%]) and 78% (95% CI [69–86%]). The diagnostic odds ratio was estimated at 5.47 (95% CI [2.86–10.46]). Three studies compared the diagnostic accuracies of PCT and CRP. The pooled sensitivity and specificity values for PCT were 57% (95% CI [26–83%]) and 75% (95% CI [68–82%]), and those for CRP were 67% (95% CI [35–88%]) and 73% (95% CI [69–77%]). The pooled sensitivity and specificity of PCT and CRP did not differ significantly (*p* = 0.61 and *p* = 0.63). The diagnostic accuracy of PCT was similar to that of CRP as measured by the area under the HSROC curve (0.73, CI = 0.61–0.91 vs. 0.74, CI = 0.61–0.95, *p* = 0.93).

**Conclusion:**

While elevated PCT levels can be indicative of potential infection, they should not be solely relied upon to exclude infection. We recommend not using the PCT test in isolation; Instead, it should be carefully interpreted in the context of clinical findings.

**Supplementary Information:**

The online version contains supplementary material available at 10.1186/s12879-024-09174-7.

## Introduction

Infection presents a substantial threat to individuals with cancer, magnified by their increased susceptibility resulting from compromised immune reactions and intricate relationships between immune suppression and cancer development [1]. The compromised immune system, often a consequence of malignancies and chemotherapy or targeted therapies, creates an environment conducive to opportunistic infections [1]. The complicated tumor microenvironment, characterized by immune cell dysfunction and cytokine imbalances, further contributes to the complexity of infection-related complications [2]. Managing infections in this vulnerable population requires timely diagnosis and comprehensive approaches that consider the complex interaction between cancer biology, immunology, and infectious disease dynamics.

Fever in cancer patients can result from a variety of non-infectious causes, including tumor-related inflammation, chemotherapy-induced fever, and radiation therapy effects. The overuse of antibiotics in non-infectious fever cases in cancer patients can lead to several negative impacts. First, it can contribute to the development of antibiotic resistance [3]. Over time, this can limit treatment options for bacterial infections that may arise during the course of cancer treatment, when patients are already immunocompromised [4]. Second, the unnecessary use of antibiotics can disrupt the patient’s microbiome, leading to gastrointestinal issues and other complications. This disruption may weaken the patient’s overall health and make them more vulnerable to infections in the long run [5, 6].

Procalcitonin (PCT) has garnered attention as a potential diagnostic biomarker for infection in cancer patients. PCT serves as a pivotal tool for distinguishing infection-induced inflammation from non-infectious complications in general population [7]. By aiding in the early identification of bacterial infection, a pressing concern for immunocompromised cancer patients, PCT has the potential to enable timely interventions and improve clinical outcomes in these patients. Some systematic reviews and meta-analyses have been conducted on the diagnostic accuracy of PCT for infection in febrile neutropenia [7–11], but there is a notable lack of such analyses specifically targeting non-neutropenic cancer patients. Therefore, we herein systematically examined the diagnostic accuracy of PCT and compared it with C-reactive protein (CRP) in this population.

## Materials and methods

The primary objective of this study was to investigate the diagnostic performance of PCT in detecting infections among adult cancer patients across existing research and to assess the findings in comparison to those of CRP when applicable data were available. In accordance with Preferred reporting items for a systematic review and meta-analysis of diagnostic test accuracy studies (PRISMA-DTA) guidelines, the Cochrane Handbook for Systematic Reviews of Diagnostic Test Accuracy, and current diagnostic accuracy review guidelines [12–14], two reviewers (Y.-C. L. and H.-T. Y.) independently identified potentially relevant studies. Subsequently, each study underwent a comprehensive review based on predefined eligibility criteria, with the inclusion of a meticulous evaluation using the PRISMA-DTA checklist. Data extraction and assessment of the methodological quality of the included studies were also conducted in line with the outlined guidelines. If disagreements occurred between the two researchers, a senior reviewer (C.-C. Y.) was consulted to adjudicate and resolve the disagreement. The study protocol was registered with PROSPERO (CRD42023421406).

### Data sources and searches

A systematic literature search was performed in MEDLINE (OvidSP), EMBASE, and Cochrane Central Register of Controlled Trials (CENTRAL) to identify all relevant diagnostic accuracy studies published before 20 June 2023. We selected medical subject headings (MeSH) and keywords to capture the concepts of procalcitonin, neoplasm, and infection (Appendix Table 1). We put no restriction on the time, location, and language of our search at this step. The list of references of each primary study was also checked to identify additional relevant studies.

**Table 1:** Main characteristics of the included studies

Author	Year	Country	Study design	Sample size	Prevalence (infection)	Mean age, years (range)^†^	Biomarkers studied	Underlying cancer	Diagnostic criteria for infection	Inclusion criteria	PCT assays	PCT collection time
Vassallo	2021	France	RS	131	66.4%	67.9 ($\pm$12.4)	PCT, CRP/PCT	Solid tumors	MDI or CDI	Fever and active solid tumors, with 2 patients with severe neutropenia	Not reported	The day of admission
Ding	2020	China	RS	588	52.9%	60.6 ($\pm$40.0)	PCT, CRP, WBC, NEU, NLR	Lung cancer	MDI (virus, fungus, and parasite excluded)	Lung cancer patients with non-neutropenic fever	Serum level via immunofluorescent assay (COBAS E602 analyzer)	Within 48 h of fever
Blouin	2020	USA	RS	2031 (episodes)	16.3%	62 (19–96)	PCT	Solid and hematologic cancers	BSI	Adult cancer patients with suspected sepsis, non-neutropenic subgroup	Serum level via immunofluorescent assay (VIDAS ® B-R-A-H-M-S PCT ™ (bioMerieux))	Within 6 days of fever
Yang	2019	Korea	RS	341	5.3%	53.5 ($\pm$12.2)	PCT, CRP	Hematologic cancers	Bacteremia	Patients with hematological malignancies and febrile episode, non-neutropenic subgroup	Serum level via immunofluorescent assay (ADVIA Centaur B.R.A.H.M.S PCT)	Fever within 24 h
Zhao	2018	China	RS	49	44.7%	Median 68 (53–80)	PCT, CRP	NSCLC	BSI	A diagnosis of NSCLC, axillary temperature > 37.5 °C, and the absence of neutropenia	Serum level via immunofluorescent assay (brand name not reported)	NR
Penel	2001	France	PS	62	69.4%	NR	PCT, CRP	Solid tumors	MDI or CDI	Febrile patients with solid tumor and no neutropenia	Serum level via chemoluminescent immunoassay kit (LUMItest; Brahms Diagnostica, Berlin, Germany)	The day of admission
Kallio	2000	Finland	PS	66	84.8%	57 ($\pm$14.8)	PCT, CRP, IL-8	Solid and hematologic cancers	MDI or CDI	Cancer patients with suspected infection, 7 of whom (11%) have neutropenia	Serum level via chemoluminescent immunoassay kit (LUMItest; Brahms Diagnostica, Berlin, Germany)	The day of admission

NR: non reported; PS: prospective studies; RS: retrospective studies; PCT: procalcitonin; CRP: C-reactive protein; MDI: microbiologically documented infection; CDI: clinically documented infection; WBC: white blood cell; NEU: neutrophil; NLR: neutrophil-to-lymphocyte ratio; BSI: bloodstream infection, IL: interleukin; NSCLC: non-small cell lung cancer

^†^ When the mean and standard deviation of the age of the whole group were not reported, we calculated these values by combining two groups sequentially using Cochrane’s formula

### Study selection

Articles meeting the following criteria were eligible for review: (1) prospective or retrospective diagnostic studies, (2) involving adult patients over the age of 18 with solid or hematological malignancies, (3) conducted in hospital settings (emergency departments, wards, or intensive care units), and (4) utilizing PCT for the detection of infection. We excluded case reports, case series with a sample size of less than 10, animal studies, pediatric studies, studies where sensitivity and specificity of PCT were not provided or could not be derived from 2 × 2 tables, and studies which included more than 15% of neutropenic patients. Studies with combined adult and child populations were also excluded if the outcome data for adult patients could not be reported separately. Two reviewers (Y.-C. L. and H.-T. Y.) independently screened all studies by title and abstract using EndNote 20 (Clarivate Analytics, Philadelphia, PA). Full-text articles were obtained for all included abstracts and reviewed by the same two reviewers. To assess the reliability of the studies’ eligibility criteria, we applied the inclusion criteria to a randomly selected 10% of all articles during the full-text review stage. Discrepancies were resolved by consulting a third reviewer (C.-C. Y.). Interobserver agreement between the two authors was assessed by calculating Cohen’s kappa statistic.

### Data extraction and quality assessment

Data extraction and assessment of the risk of bias were performed by the same two reviewers using a standardized data extraction form, and disagreements were resolved through consensus or recourse to a third reviewer (C.-C. Y.). General data items extracted from the studies included characteristics of the studies, geographical location, participant inclusion/exclusion criteria, characteristics of patients, cutoff values of the index test (i.e., PCT and CRP), and reference standards [i.e., microbiologically documented infection (MDI), clinically documented infection (CDI), bloodstream infection (BSI), and bacteremia]. Each reviewer also recorded the values of true-positive (i.e., a diagnosis of infection using PCT or CRP, and confirmed by the reference standard (including MDI, CDI, BSI, and bacteremia)), false-positive, true-negative, and false-negative, as well as the sensitivity and specificity values of PCT, along with CRP when available. The risk of bias for each of the included studies was evaluated with the Quality Assessment of Diagnostic Accuracy Studies 2 (QUADAS-2) tool, consisting of two components: risk of bias and concerns regarding applicability using four domains of bias and applicability—patient selection, index test, reference standard, and flow and timing [15]. No specific eligibility criterion was established for the reference standard. The reference standards employed by each study are detailed as part of the overall study characteristics. This approach sought to assess the methodological quality, reporting, and validity of the included studies, ensuring a thorough evaluation of diagnostic test accuracy studies.

### Data synthesis and analysis

We determined the sensitivity and specificity for each study by constructing a 2 × 2 contingency table. When multiple sensitivity or specificity values were reported in a single study, we selected either the predefined value or, if not reported, the pair of sensitivity and specificity values that maximized the Youden index *(J = Sensitivity−(1 − Specificity))*. For the diagnostic accuracy meta-analysis, we employed a hierarchical summary receiver operator characteristic (HSROC) model, a method which allows for both fixed and random effects relating to threshold and accuracy. The HSROC model was utilized to ascertain the summary points of various accuracy parameters including sensitivity, specificity, positive and negative likelihood ratios, and diagnostic odds ratios [16]. Given diverse cutoff values in our analyzed studies, the HSROC model’s capacity to illustrate the sensitivity and 1-specificity relationship became particularly valuable. The model’s strength lies in accommodating this correlation, effectively facilitating heterogeneity investigation between studies [17, 18]. The 95% confidence region and the 95% prediction region encompassing the pooled estimates were graphically depicted to illustrate the precision of the pooled value estimations (confidence ellipse around the mean value) and to illustrate the amount of between-study variation (prediction ellipse). We assessed heterogeneity through a visual examination of both sensitivity and specificity estimates on forest plots and receiver-operating characteristic (ROC) space. We further performed predefined subgroup analyses to evaluate the heterogeneity among studies stratified by the number of patients ($$ \ge $$ 131 or $$ <$$ 131), reference standard (BSI or non-BSI), study design (prospective or retrospective), region (Asia or Non-Asia), prevalence ($$ \ge $$ 52.9% or $$ <$$ 52.9%), and cutoff value ($$ \ge $$ 0.5 mg/L or $$ <$$ 0.5 mg/L). The median value of specific continuous variables in the included studies was used to divide them into different subgroups. We conducted three sensitivity analyses to determine the robustness of the meta-analyses. First, we removed studies that were not entirely non-neutropenic (i.e., some studies included a few neutropenic patients). Second, we removed studies in which the PCT collection time was more than 24 h from admission. Third, we performed a leave-one-out analysis by removing each study in turn and reanalyzing the data. We intended to assess publication bias by utilizing the Deek’s effective sample size funnel plot alongside the log diagnostic odds ratio. The Fagan’s nomogram was used to visually assess diagnostic performance. All pooled meta-analytic statistics were reported with their corresponding 95% confidence intervals. Statistical and meta-analyses were conducted in STATA version 17 (Stata Corporation, College Station, TX, USA) using several modules: Metandi for summary estimates and HSROC plots, Metadta for forest plots, and Midas for Fagan’s nomogram. When the pooled study number was less than four in subgroup analyses and beyond STATA’s processing capability, summary estimates were obtained with R version 4.1.2 (R Foundation for Statistical Computing, Vienna, Austria).

## Results

### Search results

**Fig. 1:**
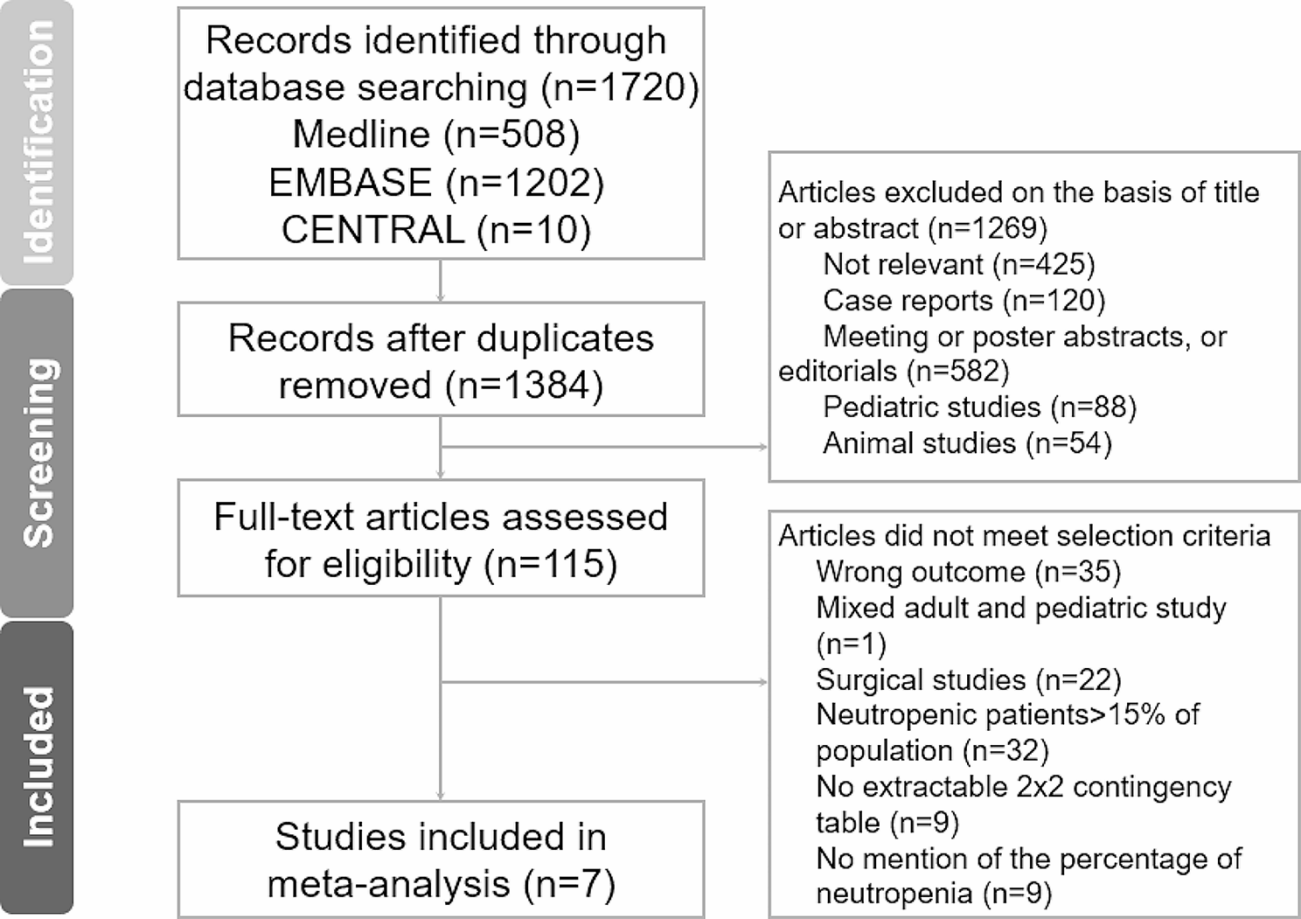
Flow chart of study identification, screening, inclusion, and exclusion for meta-analysis

Our database search returned 1,720 articles. After the initial screening, 1,605 articles were excluded. One hundred and fifteen articles were subjected to further full-text reviews, and 108 were excluded, leaving seven for inclusion (Fig. 1) [21–25]. The search in the reference lists of the identified articles did not return any more eligible studies. The percent agreement between the two reviewers for article selection was 90%, and Cohen’s kappa was k = 0.78.

### Study characteristics

Table 1 presents the characteristics of the included studies. All eligible studies were published between 2000 and 2021, the median sample size is 131 (interquartile range: 64–465), and the final analysis included a total of 3,266 patients. For the geographic area, three (42.9%) were conducted in Europe [19, 20, 25], three (42.9%) were conducted in Asia [21, 22, 24], and one (14.3%) was conducted in the United States [23]. For the study design, two studies (28.6%) were prospective cohort studies [19, 20], and five (71.4%) were retrospective cohort studies [21–25]. No case–control studies or randomized controlled trials were included. For the collected sample of procalcitonin, only one study did not specify the type of sample used [25]. In contrast, other studies employed the measurement of procalcitonin in serum. Among all 3,266 patients, 868 (26.6%) patients were in the infection group, and 2,398 (73.4%) were in the control group. The percentage of patients with infection ranged from 5.3 to 84.8% among the studies. The PCT cutoff value for detecting infection ranged from 0.105 (mg/L) to 1.695 (mg/L), with a median value of 0.5 (mg/L). Two studies included some neutropenic patients: one study included two patients (1.5%) [25], and another comprised seven (11%) of the total population [19]. Regarding the reference standard for infection, four studies (57.1%) defined infection as MDI or CDI [19, 20, 24, 25], two studies (28.6%) classified it as BSI [21, 23], and one study (14.3%) identified it as bacteremia [22].

### Quality assessment

**Fig. 2:**
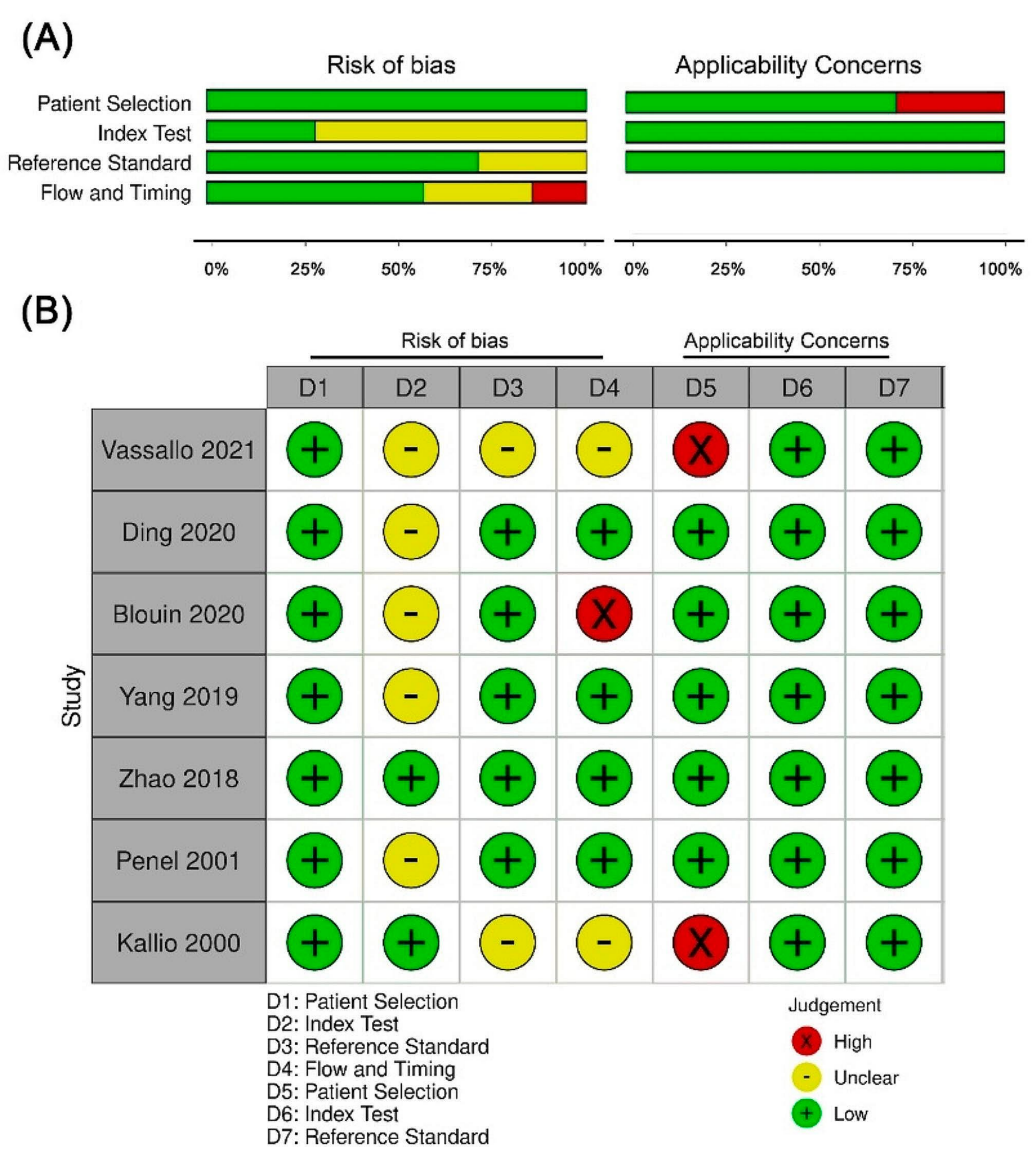
Quality assessment for seven studies (QUADAS-2)

The quality assessments based on the QUADAS-2 criteria are succinctly outlined in Fig. 2. Within the patient selection domain, all studies were deemed to carry a low risk of bias due to their comprehensive descriptions of enrollment design. In the domain of the index test, five (71.4%) studies were associated with an unclear risk of bias since they calculated sensitivity and specificity using the optimal cutoff value other than the predefined value [20, 22–25]. In the reference standard domain, two (28.5%) studies had an unclear risk of bias because CDI may be subjectively determined by healthcare providers and cannot be confirmed to be unaffected by the index test [19, 25]. Moving to the flow and timing domain, one study (14.3%) exhibited a high risk of bias because the time interval of PCT collection is within six days, which encompasses a broad range and made it challenging to predict whether it is an appropriate time interval [23]. Additionally, two studies presented an unclear risk of bias in this domain, attributed to the usage of different reference standards among the patients [19, 25]. Regarding applicability, two studies displayed a high risk in the patient selection domain as they enrolled not only non-neutropenic patients but also those with neutropenia [19, 25]. All other studies were categorized as having a low risk in this aspect.

### Primary analysis of overall accuracy

**Fig. 3:**
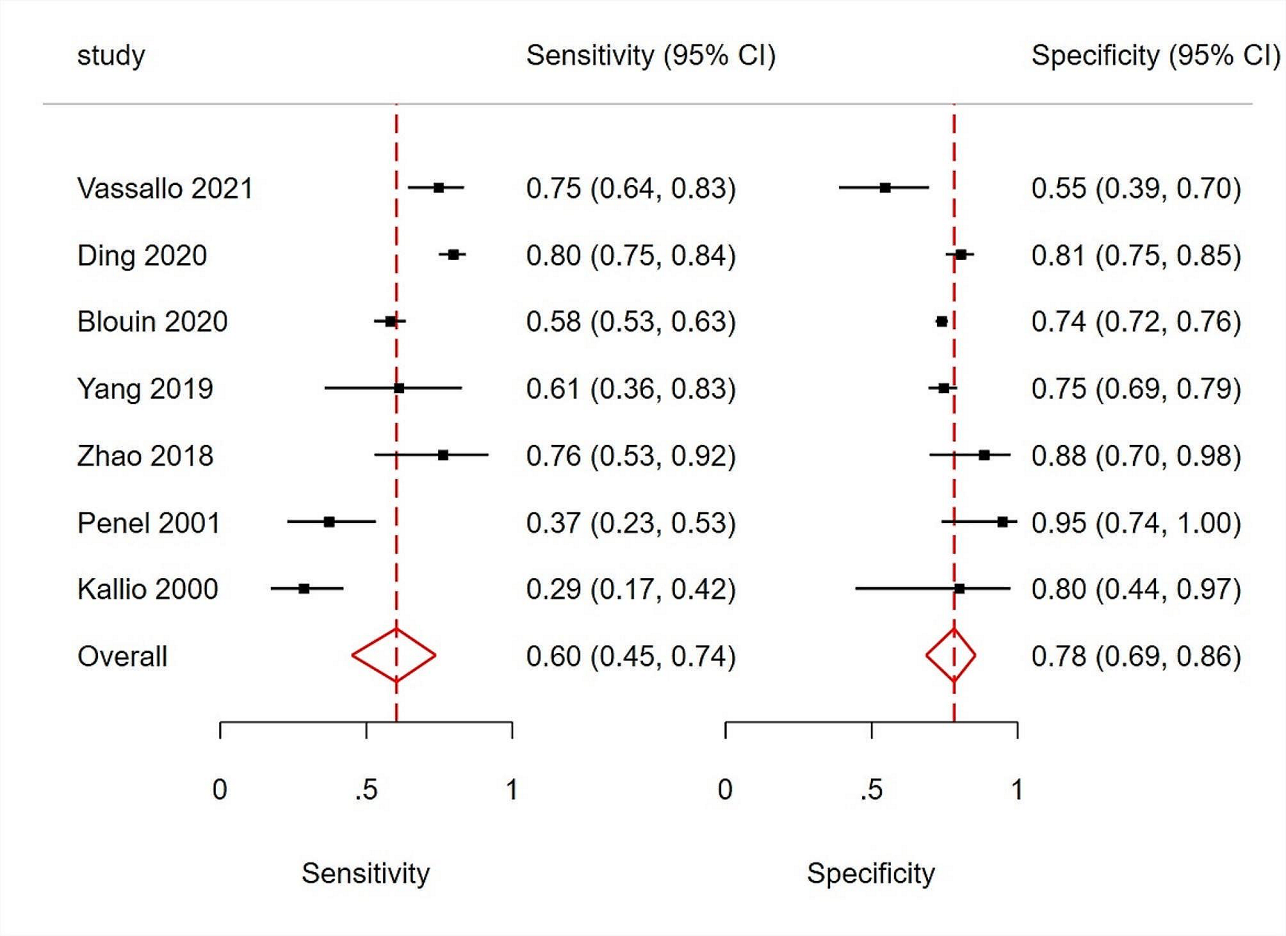
Forest plots of the sensitivity and specificity for procalcitonin across all included studies

**Fig. 4:**
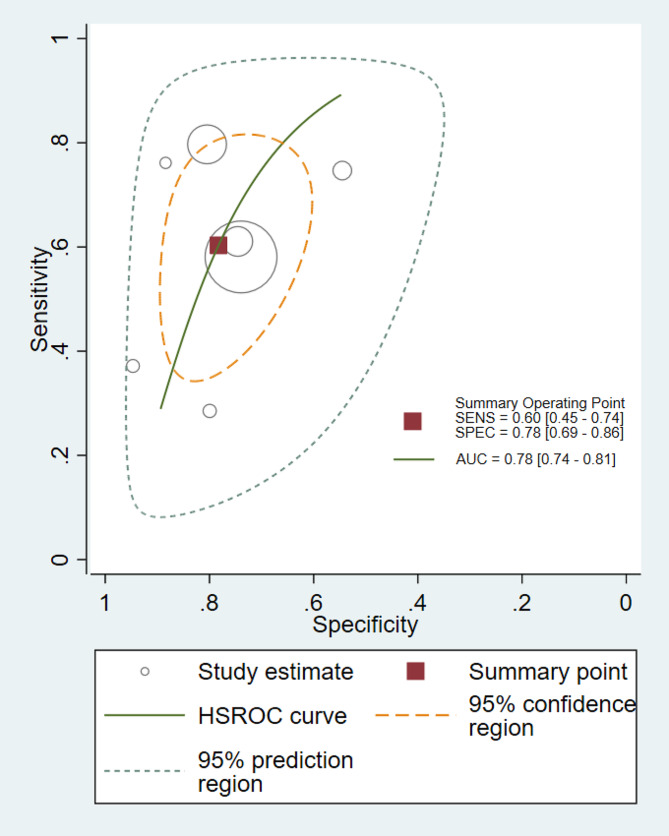
Hierarchical summary receiver operating characteristic plot of procalcitonin across all included studies

Figure 3 shows the forest plots for the sensitivity and specificity of PCT reported in the seven included studies. The pooled sensitivity across all studies was 60% (95% CI [45–74%]), and the pooled specificity was 78% (95% CI [69–86%]). The estimated diagnostic odds ratio was 5.47 (95% CI [2.86–10.46]). The pooled estimates of positive and negative likelihood ratios were 2.77 (95% CI [1.89–4.70]) and 0.51 (CI [0.36–0.72]), respectively. The HSROC curves, together with the bivariate summary points of specificity and sensitivity and their 95% confidence regions are shown in Fig. 4. The area under the HSROC curve was 0.78 (95% CI [0.74–0.81]). Consistent with many meta-analyses of diagnostic accuracy studies, we observed substantial heterogeneity among the included studies. This was evidenced by the wide variation in sensitivity and specificity estimates among them.

### Subgroup analysis, sensitivity analysis, publication bias, and Fagan’s nomogram

**Fig. 5:**
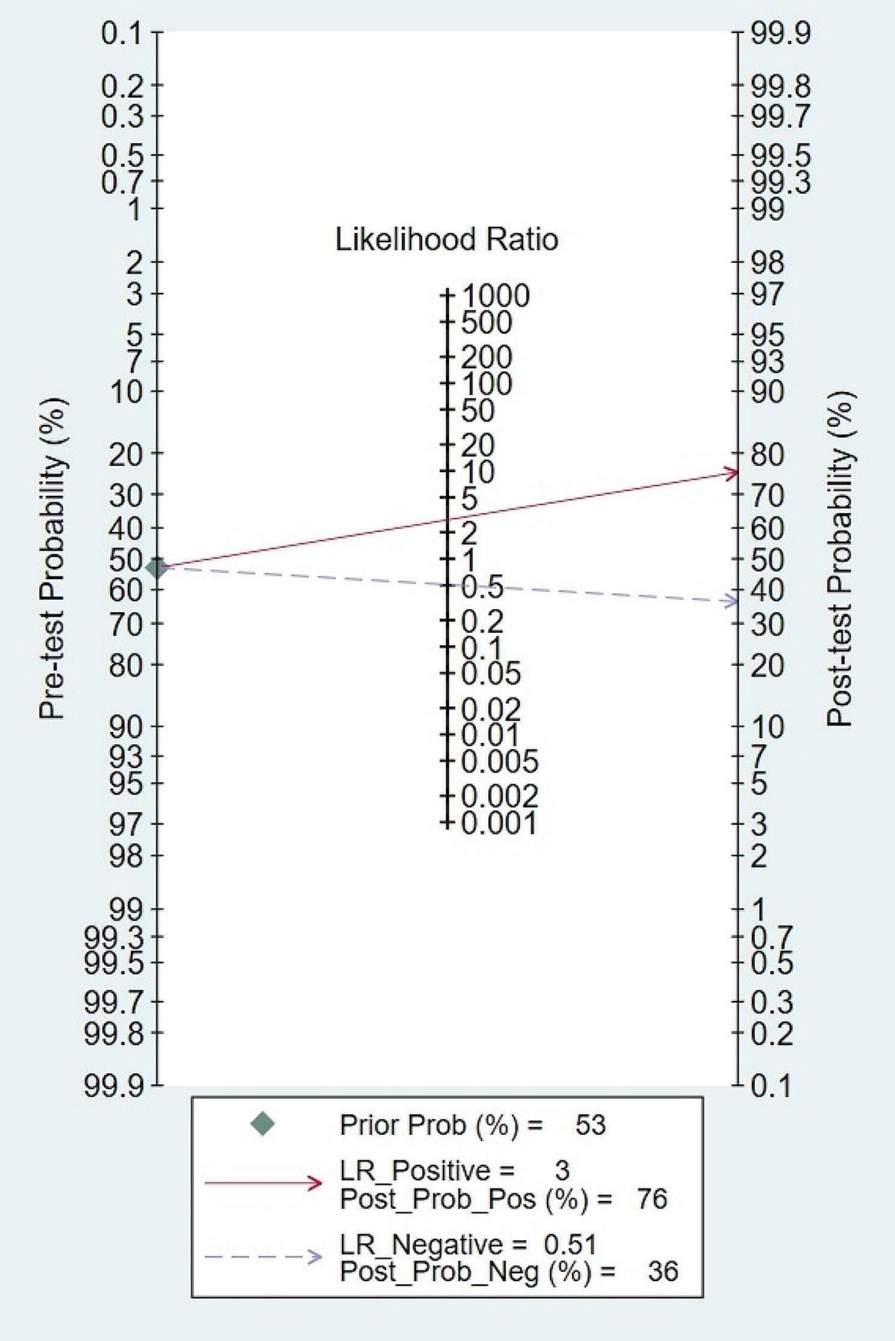
Fagan’s nomogram for procalcitonin

We performed subgroup analyses to analyze sources of heterogeneity (Table 2). Studies with $$ \ge $$ 131 patients had a significantly lower pooled specificity (76%, Cl = 73–79% vs. 89%, CI = 80–97%, *p* < 0.01) than those with $$ <$$ 131 patients. Studies with a prospective design had a significantly lower pooled sensitivity (32%, Cl = 20–48% vs. 71%, CI = 62–78%, *p* < 0.01) than those with a retrospective design. Studies with a PCT cutoff value $$ \ge $$ 0.5 mg/L had a significantly lower pooled sensitivity (52%, CI = 39–65% vs. 77%, CI = 58–89%, *p* < 0.01) than those with a cutoff value < 0.5 mg/L. The pooled sensitivity and specificity were not significantly different among the reference standard (BSI or Non-BSI), region (Asia or Non-Asia), and prevalence ($$ \ge $$ 52.9% or $$ <$$ 52.9%). A substantial degree of heterogeneity existed among most subgroups. The sensitivity analysis of PCT demonstrated that the area under the HSROC curve did not exhibit significant differences when including only studies with entirely non-neutropenic populations, those with PCT collection time within 24 h of admission, or upon removal of any single study (Appendix Table 2). Publication bias was not assessed because fewer than 10 studies were included [26]. For clinical utility evaluation, we assumed a pre-test probability of 52.9% (i.e., median value of prevalence of included studies). The Fagan’s nomogram for PCT showed a post-test probability of 76% positive and 36% negative (Fig. 5).

**Table 2:** Summary of subgroup analysis of procalcitonin in the diagnosis of infection

Subgroup	Number of studies	Pooled sensitivity (95% CI)	Heterogeneity I^2^(%)	Subgroup difference P value	Pooled specificity (95% CI)	Heterogeneity I^2^(%)	Subgroup difference P value	AUC (95% CI)
**Sample size**								
Number of patients $\ge$ 131	4	0.69(0.58,0.81)	92	0.19	0.76(0.73,0.79)	79	< 0.01^*^	0.77(0.63,1.00)
Number of patients $$ <$$ 131	3	0.44(0.27,0.62)	84		0.89(0.80,0.97)	0		0.73(0.60,0.94)
**Reference standard**								
BSI	3	0.65(0.42,0.87)	22	0.89	0.77(0.66,0.88)	24	0.14	0.76(0.59,1.00)
Non-BSI	4	0.57(0.38,0.77)	96		0.79(0.67,0.91)	81		0.74(0.65,0.86)
**Study design**								
Prospective	2	0.32(0.20,0.48)	0	< 0.01^*^	0.90(0.72,0.97)	26	0.09	0.67
Retrospective	5	0.71(0.62,0.78)	89		0.76(0.73,0.79)	76		0.79(0.72,0.87)
**Region**								
Asia	3	0.78(0.66,0.91)	41	0.22	0.80(0.69,0.91)	57	0.18	0.89(0.87,0.93)
Non-Asia	4	0.51(0.36,0.66)	91		0.79(0.68,0.90)	74		0.71(0.65,0.80)
**Prevalence**								
$\ge$ 52.9%	4	0.57(0.38,0.77)	96	0.52	0.79(0.67,0.91)	81	0.28	0.74(0.65,0.86)
$$ <$$ 52.9%	3	0.65(0.42,0.87)	22		0.77(0.66,0.88)	24		0.76(0.59,1.00)
**Cutoff value (mg/L)**								
$\ge$ 0.5	5	0.52(0.39,0.65)	88	< 0.01^*^	0.77(0.68,0.85)	65	0.13	0.71(0.65,0.79)
$$ <$$ 0.5	2	0.77(0.58,0.89)	0		0.83(0.69,0.91)	0		0.88(0.64,1.00)

BSI: bloodstream infection; CI: confidence interval; AUC: Area under the curve

^*^P < 0.05

### Head-to-head comparison of the performances of PCT and CRP

Three of the seven studies directly compared the diagnostic accuracies of PCT and CRP (Table 3 and Appendix Table 3). The pooled sensitivity and specificity values for PCT were 57% (95% CI [26–83%]) and 75% (95% CI [68–82%]), and those for CRP were 67% (95% CI [35–88%]) and 73% (95% CI [69–77%]). The pooled sensitivity and specificity of PCT and CRP did not differ significantly (*p* = 0.61 and *p* = 0.63). The diagnostic accuracy of PCT was similar to that of CRP as measured by the area under the HSROC curve (0.73, CI = 0.61–0.91 vs. 0.74, CI = 0.61–0.95, *p* = 0.93) (Appendix Fig. 1).

**Table 3:** Summary of diagnostic accuracy of procalcitonin and C-reactive protein in detecting infection

Author, year	Infection/control (n)	Procalcitonin				C-reactive protein			
		Sensitivity	Specificity	AUC	Cutoff^†^ (mg/L)	Sensitivity	Specificity	AUC	Cutoff (mg/L)
Vassallo et al. 2021	87/44	0.75	0.55	–	0.52	–	–	–	–
Ding et al. 2020	311/277	0.80	0.80	0.87	0.105	0.86	0.71	0.86	12.2
Blouin et al. 2020	332/1699	0.58	0.74	0.71	1.695	–	–	–	–
Yang et al. 2019	18/323	0.61	0.75	0.76	0.5	0.67	0.74	0.76	100
Zhao et al. 2018	21/26	0.76	0.89	0.84	0.44	–	–	–	–
Penel et al. 2001	43/19	0.37	0.95	–	1	–	–	–	–
Kallio et al. 2000	56/10	0.29	0.80	0.61	0.5	0.39	0.70	0.42	140

AUC: Area under the curve; −: Not available

^†^Yang et al. and Kallio et al. employed predefined cutoffs, whereas Ding et al. and Blouin et al. determined their cutoffs using Youden’s index. Vassallo et al., Penel et al., and Zhao et al. utilized optimal cutoffs, although without a precise definition

## Discussion

This is the first systematic review and meta-analysis to evaluate the diagnostic accuracy of PCT in the detection of infections among non-neutropenic cancer patients. Our study reveals that PCT serves as a biomarker with moderate specificity but relatively poor sensitivity in distinguishing infections within this patient population. Therefore, while PCT can be considered indicative of potential infection, it should not be relied upon as a sole biomarker for excluding infection. Detecting infections in cancer patients is challenging due to the subtle or hidden nature of their symptoms. Several studies have been dedicated to identifying reliable biomarkers for determining infection in cancer patients. Phillips et al. performed the first systematic review to assess the predictive value of biomarkers of inflammation and infection in pediatric cancer patients with febrile neutropenia in 2012 [8]. The review indicated that IL-6, IL-8, and PCT appear promising in predicting significant infection [8]. Since then, a plethora of related literature has emerged with time. The latest review is an updated review of 17 studies conducted by Arif et al. in 2019, highlighting the significant role of PCT in discriminating infections in pediatric cancer patients with febrile neutropenia [9]. Nonetheless, prior reviews have predominantly focused on neutropenic fever in pediatric cancer patients, with only one study addressing the role of PCT in adult neutropenic fever [7–11], leaving the realm of the non-neutropenic population relatively understudied [27]. Among the array of inflammatory markers, the diagnostic accuracy of PCT and CRP in bacterial infection and sepsis has been extensively studied [28, 29]. A meta-analysis by Tan et al. revealed that the pooled specificity of PCT for sepsis diagnosis was superior to that of CRP [30]. Additionally, since CRP levels in cancer patients can be influenced by inflammatory responses from tumor cells, they may not accurately indicate the presence of an infection [31]. Thus, the clinical significance of PCT in non-neutropenic cancer patients warrants substantial attention, especially for frontline physicians.

Our meta-analysis has a distinct strength as it represents the first study to investigate the diagnostic accuracy of PCT for infection among non-neutropenic cancer patients through a systematic review and meta-analysis. In addition, our findings achieve heightened reliability due to the comprehensive evaluation of bias risk utilizing the QUADAS-2 tools. Furthermore, we meticulously analyzed potential heterogeneity among the included studies by conducting various subgroup analyses and ensured the robustness of our findings through sensitivity analyses.

In our study, PCT demonstrated a pooled sensitivity of 60% and a pooled specificity of 78%. PCT measurement had a moderate rule-in value, but a poor rule-out value, for diagnosis of infection among non-neutropenic cancer patients. The moderate rule-in value suggests that elevated PCT levels can be indicative of infection in non-neutropenic cancer patients, offering clinicians useful, though not definitive, insights for considering infectious conditions. However, the observed poor rule-out value implies that normal or low PCT levels may not reliably rule out the presence of infection. In comparison, the systematic review and meta-analysis conducted by Wu et al. focusing on adult febrile neutropenia reported similar results, with pooled sensitivity and specificity for diagnosing bacterial infection as 0.65 (95% CI 0.55–0.73) and 0.79 (95% CI 0.71–0.85), respectively [10]. The consistent inadequate performance of PCT suggested that PCT measurement should not be solely relied on for excluding infections in both patient populations.

Heterogeneity analyses demonstrated significant variability in sensitivity and specificity across the included studies. This underscores the complex nature of infection in cancer patients and highlights the need for careful interpretation of our results. Variations in reference standard, geographic region, and prevalence did not significantly affect the pooled sensitivity and specificity. Studies with a larger patient population exhibited lower pooled specificity compared to those with fewer patients. In larger-scale studies, the inclusion of a diverse patient population introduces greater variability in patient characteristics, cancer stages, and potential confounding factors. In contrast, smaller-scale studies focus on specific patient groups and tend to exhibit more consistency. The choice of PCT cutoff value appeared to influence diagnostic sensitivity. A previous study had suggested a cutoff value of 0.5 mg/L to be the most helpful biochemical parameter in detecting severe infection, mainly bloodstream infection, in patients with hematological cancers [32]. In our meta-analysis, studies using a cutoff value ≥ 0.5 mg/L showed a lower pooled sensitivity than those with a lower cutoff value. Furthermore, studies with a prospective design demonstrated a lower pooled sensitivity compared to those with a retrospective design. Differences in pooled sensitivity between the two study designs can be attributed to differences in data collection and analysis methods. Prospective studies involve real-time data collection as disease progression or treatment unfolds, offering an authentic depiction of patient characteristics but providing less control over study conditions. Conversely, retrospective studies rely on historical data from medical records, allowing researchers to carefully select and analyze data points in accordance with their research questions, which can potentially introduce bias into the diagnostic accuracy of PCT. Sensitivity analyses, including studies with exclusively non-neutropenic populations or removing individual studies, did not result in significant differences in the area under the HSROC curve. This suggests that the overall performance of PCT remained relatively consistent, regardless of the inclusion or exclusion of neutropenic patients. The direct comparison of diagnostic accuracies between PCT and CRP within our study provides insights into the relative performance of these two biomarkers in non-neutropenic cancer patients. Three of the seven studies included in our analysis specifically examined the diagnostic utility of PCT and CRP. Our analysis found no significant differences in diagnostic performance between PCT and CRP. However, due to the limited studies available for comparison, it is imperative that more large-scale prospective investigations are required to compare the diagnostic accuracy of PCT and CRP in differentiating infections among non-neutropenic cancer patients.

Our study has certain limitations. First, the notable variability in inclusion criteria among the studies can impact the reported infection prevalence, resulting in a wide variation ranging from as low as 5% to as high as 85%. This diversity indicates substantial differences in populations and settings, further contributing to the overall heterogeneity observed in our meta-analysis. Second, factors such as variations in PCT collection time, PCT test assays, study design, and reference standard across different studies introduce heterogeneity as well. Among the studies included, 2 utilized the old-generation PCT test (LUMItest; Brahms Diagnostica, Berlin, Germany) [19, 20]. Even though the old-generation PCT test (LUMItest) can detect PCT levels down to 0.5 ng/mL, results in the range below 0.5 ng/mL may not be very precise [33]. Despite our efforts to conduct subgroup analyses, these differences might still introduce bias into our results. Third, discrepancies in recommended PCT cutoffs by different manufacturers, along with studies not adhering to predefined values, require further investigation to establish optimal thresholds. Fourth, it is crucial to note that our comparison focused only on studies with simultaneous measurements of PCT and CRP. Articles solely assessing CRP were not included, potentially impacting the interpretation of CRP performance. More studies are necessary to further increase the credibility of our findings. Last, the variability in the selection of cancer patient features and reference standards among included studies potentially restricts the applicability of our findings across various clinical conditions.

## Conclusion

In conclusion, our findings reveal that PCT serves as a biomarker with moderate specificity but relatively poor sensitivity. While elevated PCT levels may indicate potential infection, they should not be solely relied upon to exclude infection and withhold antibiotics. We recommend not using the PCT test in isolation; Instead, it should be carefully interpreted in the context of clinical findings. To advance clinical decision-making and enhance patient care in this vulnerable population, future large-scale studies are warranted to provide stronger evidence for the optimal use of PCT in the diagnosis of infection among non-neutropenic cancer patients.

### Electronic supplementary material

Below is the link to the electronic supplementary material.

Appendix 1

## Data Availability

No datasets were generated or analysed during the current study.

## Abbreviations

PCT: procalcitonin
CRP: C-reactive protein
HSROC: hierarchical summary receiver operator characteristic
CI: confidence interval
PRISMA: Preferred Reporting Items for Systematic Reviews and Meta-Analyses
MeSH: medical subject headings
MDI: microbiologically documented infection
CDI: clinically documented infection
BSI: bloodstream infection
QUADAS-2: Quality Assessment of Diagnostic Accuracy Studies 2
