# Supplementary material for: Diagnostic accuracy of procalcitonin in adult non-neutropenic cancer patients with suspected infection: a systematic review and meta-analysis
Source: BMC Infect Dis. 2024 Mar 4;24:278. doi: 10.1186/s12879-024-09174-7 (PMC10910706; doi:10.1186/s12879-024-09174-7)

# Appendix table 1. Electronic search strategies

**MEDLINE (OvidSP)**

Date of search: June 20, 2023

|  | **Search strategy** | **Results** |
| --- | --- | --- |
| 1 | exp Neoplasms/ | 3843088 |
| 2 | Infections/ | 40683 |
| 3 | infection$.mp. | 2095823 |
| 4 | (cancer$ or neoplas$ or oncolog$ or malignan$ or tumo?r$ or sarcoma$ or leukaemi$ or leukemi$ or chemotherap$).ti,ab. | 3540631 |
| 5 | procalcitonin.mp. | 7363 |
| 6 | (procalcitonin or pro-calcitonin).ti,ab. | 7224 |
| 7 | 2 or 3 | 2095823 |
| 8 | 1 or 4 | 4605731 |
| 9 | 6 and 7 and 8 | 508 |

**EMBASE**

Date of search: June 20, 2023

|  | **Search strategy** | **Results** |
| --- | --- | --- |
| 1 | 'neoplasms'/exp | 5999257 |
| 2 | 'cancer':ab,ti OR 'neoplas*':ab,ti OR 'oncolog*':ab,ti OR 'malignan*':ab,ti OR 'tumor*':ab,ti OR 'tumo$r*':ab,ti OR 'sarcoma*':ab,ti OR 'leukaemi*':ab,ti OR 'leukemi*':ab,ti OR 'chemotherap*':ab,ti | 5562776 |
| 3 | 'infections'/de | 337904 |
| 4 | 'infection$':ab,ti | 2214104 |
| 5 | 'procalcitonin':ab,ti OR 'pro-calcitonin':ab,ti | 14627 |
| 6 | #1 OR #2 | 7172605 |
| 7 | #3 OR #4 | 2323024 |
| 8 | #5 AND #6 AND #7 | 1202 |

# Appendix table 2. Sensitivity analyses of procalcitonin using leave-one-out method

| Author, year | AUC (95% CI) | Delta AUC |
| --- | --- | --- |
| Combined | 0.78 (0.74,0.81) | - |
| Excluding studies not entirely non-neutropenic^†^ | 0.78 (0.74,0.81) | 0 |
| Excluding studies with PCT collection time of more than 24 hours^‡^ | 0.73 (0.65,0.82) | -0.05 |
| Excluding Vassallo et al. 2021 | 0.75 (0.72,0.79) | -0.03 |
| Excluding Ding et al. 2020 | 0.73 (0.69,0.77) | -0.05 |
| Excluding Blouin et al. 2020 | 0.79 (0.75,0.82) | 0.01 |
| Excluding Yang et al. 2019 | 0.78 (0.75,0.82) | 0 |
| Excluding Zhao et al. 2018 | 0.76 (0.72,0.80) | -0.02 |
| Excluding Penel et al. 2001 | 0.77 (0.73,0.80) | -0.01 |
| Excluding Kallio et al. 2000 | 0.79 (0.75,0.82) | -0.01 |

AUC: Area under the curve; −: Not available

^†^Vassallo et al. 2021 and Kallio et al. 2000

^‡^Ding et al. 2020, Blouin et al. 2020, and Zhao et al. 2018

# Appendix table 3. Diagnostic accuracy for procalcitonin and C-reactive protein in the included studies

| Biomarkers | Author, year | TP | FP | TN | FN | Sensitivity | Specificity |
| --- | --- | --- | --- | --- | --- | --- | --- |
| procalcitonin | Vassallo et al. 2021 | 65 | 20 | 24 | 22 | 0.75 | 0.55 |
|  | Ding et al. 2020 | 248 | 54 | 223 | 63 | 0.80 | 0.80 |
|  | Blouin et al. 2020 | 193 | 442 | 1257 | 139 | 0.58 | 0.74 |
|  | Yang et al. 2019 | 11 | 82 | 241 | 7 | 0.61 | 0.75 |
|  | Zhao et al. 2018 | 16 | 3 | 23 | 5 | 0.76 | 0.89 |
|  | Penel et al. 2001 | 16 | 1 | 18 | 27 | 0.37 | 0.95 |
|  | Kallio et al. 2000 | 16 | 2 | 8 | 40 | 0.29 | 0.80 |
| C-reactive protein | Ding et al. 2020 | 266 | 79 | 198 | 45 | 0.86 | 0.71 |
|  | Yang et al. 2019 | 12 | 85 | 6 | 238 | 0.67 | 0.74 |
|  | Kallio et al. 2000 | 22 | 3 | 7 | 34 | 0.39 | 0.70 |

# Appendix figure 1. Comparisons of procalcitonin (PCT) and C-reactive protein (CRP) with summary receiver operating characteristic curves and 95% confidence interval region


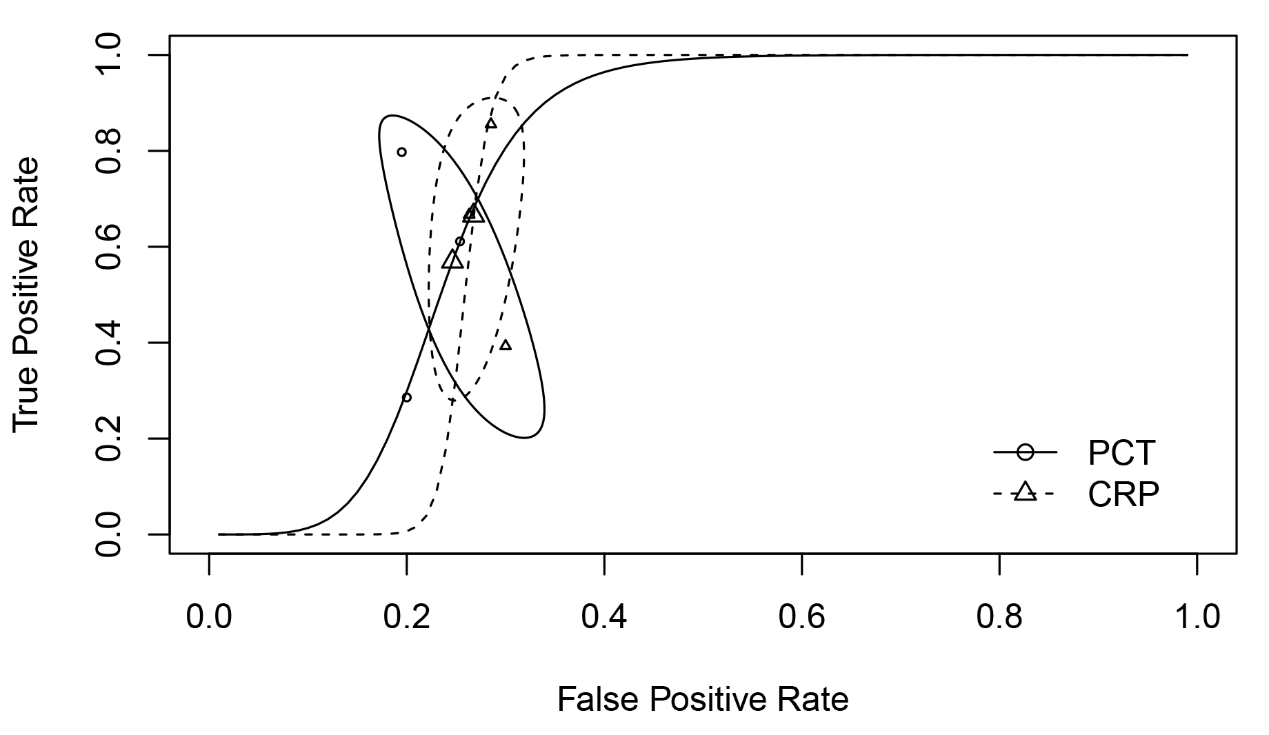

Supplement: Supplementary file 1 — Appendix 1 [file 12879_2024_9174_MOESM1_ESM.docx]
